# Anthropometric Indices in Children With Refractory Epilepsy

**Published:** 2016

**Authors:** Vahid AMINZADEH, Setila DALILI, Yalda ASHOORIAN, Shahin KOHMANAEE, Afagh HASSANZADEH RAD

**Affiliations:** 1 Pediatric Growth Disorders Research Center, 17 Th Shahrivar Hospital, School of Medicine, Guilan University of Medical Sciences, Rasht, Iran; 2 Department of Pediatrics Neurology, 17th Shahrivar Hospital, Guilan University of Medical Sciences, Rasht, Iran.; 3 Department of Pediatrics Endocrinology And Metabolism, 17th Shahrivar Hospital, Guilan University of Medical Sciences, Rasht, Iran

**Keywords:** Anthropometric indices, Obesity, Epilepsy, Children

## Abstract

**Objective:**

We aimed to assess the effect of body mass index (BMI) on reducing the risk of refractory seizure due to lipoid tissue factors.

**Materials & Methods:**

This matched case-control study, consisted of cases (Patients with refractory epilepsy) and controls (Healthy children) referred to 17 Shahrivar Hospital, Guilan University of Medical Sciences, Guilan, Iran during 2013-2014. Data were gathered by a form including demographic characteristics, type of epilepsy, predominant time of epilepsy, therapeutic approach, frequency of epilepsy, time of disease onset and anthropometric indices. We measured anthropometric indices and transformed them into Z-scores. Data were reported by descriptive statistics (mean and standard deviation) and analyzed by Pearson correlation coefficient, paired t test and multinomial regression analysis test using SPSS 19.

**Results:**

There was no significant difference between sex groups regarding anthropometric indices. Generalized and focal types of epilepsies were noted on 57.5% and 38.75% of patients, respectively. Daytime epilepsies happened in 46.25% of patients and 33.75% noted no predominant time for epilepsies. Clinicians indicated poly-therapy for the majority of patients (92.5%). The most common onset times for epilepsies were 36-72 months for 32.5% of patients. Lower onset time indicated lower frequency of refractory epilepsies. Although, there was significant difference between Zheight and predominant time of epilepsies but no significant relation was found between types of epilepsies and frequency of epilepsies with anthropometric indices. Using multivariate regression analysis by backward LR, Zweight and birth weight were noted as the predicting factors of refractory epilepsies.

**Conclusion:**

This effect may be because of leptin. Therefore, researchers recommend further investigations regarding this issue in children with epilepsy.

## Introduction

Refractory epilepsy (RE) is a neurological disease. It affects relatively 20% to 30% of epileptic patients. Clinicians commonly cannot determine the response to multiple line drug therapies in RE patients (1). Epilepsy might accompany with malnutrition and noted as two key health problems (1, 2). Malnutrition might be indicated due to chronic use of anticonvulsant drugs because they may influence food intake and energy metabolism. In addition, they can induce vomiting, anorexia and feeding difficulties in chewing or swallowing. Moreover, their energy requirement may be changed according to the impedance of the disabilities with their common activities (3). One of the most common adverse outcomes consequent of using anticonvulsants is the weight gain, although, recent investigations noted body weight gain as a result of consuming valproic acid vigabatrin, gabapentin and carbamazepine (4). Yet, there are two different hypotheses, which cause a vicious circle: malnutrition predisposes epilepsy or epilepsy predisposes malnutrition. As malnutrition can be prevented and treated, therefore, a thorough understanding of these interactions can be recommended (5). On the other hand, in animal model, the threshold of epilepsy was changed dramatically by neuropeptides especially in hippocampus (6, 7). Obesity is noted as the leading factor for maladaptive processes for exacerbating chronic diseases such as epilepsy, multiple sclerosis and Alzheimer’s disease (6, 8). On the other hand, enough endocrine products such as leptin by adipose tissue are effective in epilepsy (9, 10). The aim of the present study was to evaluate body mass index (BMI) and nutritional status in children with refractory epilepsy. Anthropometric evaluation can easily detect malnutrition; therefore, the results of this study might help to improve the treatment. It can be regarded for further researches in children with refractory epilepsy.

## Materials & Methods

This case–control study consisted of cases (Patients with refractory epilepsy) and control (healthy children) referred to 17 Shahrivar Hospital, Guilan University of Medical Sciences, Guilan, Iran during 2013-2014 .Groups were matched for age, geographical area, social and economical status. We excluded children with nutritional status impairment (Neoplasia, chronic infections), changes in energy metabolism (Hyperhypothyroidism), treated with special diets (Diabetes, phenylketonuria, celiac disease or lactose intolerance) and feeding with nasogasteric tube. All cases were treated with antiepileptic drugs during the study period. After sample size determination, we selected 80 children with refractory epilepsy from the Outpatient Clinic of Pediatric Neurology and Epilepsy in Rasht, northern of Iran, and compared with 80 healthy children without epilepsy from the Outpatient Clinic of General Pediatric. Informed consent letters were obtained from participants. Data were gathered by a form including demographic characteristics, type of epilepsy, predominant time of epilepsy, therapeutic approach, frequency of epilepsy, time of disease onset and anthropometric indices. We measured anthropometric indices and transformed them into Z-scores. Weight/age (W/A), height/age (H/A) and BMI/age (BMI/A) were assessed using WHO Anthro Plus software based on the latest WHO (2005/2007) growth charts. Data were reported by descriptive statistics (mean and standard deviation) and analyzed by Pearson correlation coefficient, paired t test and multinomial regression analysis test using SPSS 19 (Chicago, IL, USA).

**Table 1:** Anthropometric Indices in Groups

Variables	Case group N=80		Control group N=80		*P*-value
	Mean	Standard deviation	Mean	Standard deviation	
Age (mo)	98.64	30.58	99.14	30.32	*P*=0.92
Weight (kg)	29.06	11.00	31.79	10.87	*P*=0.12
height (cm)	128.66	15.32	131.02	16.18	*P*=0.34
BMI (kg/m^2^)	16.90	2.97	17.92	2.87	*P*=0.03
Z W/A*	0.17	0.97	0.78	0.98	0.001
Z H/A**	0.09	0.97	0.43	0.85	0.017
Z BMI/A***	0.18	1.20	0.69	1.07	0.005
Birth weight(kg)	3.043	0.487	3.616	3.292	*P*=0.000
Birth height	49.02	2.48	49.88	1.49	*P*=0.009

* Z W/A: Z-score for weight-for-age;

** Z H/A: Z-score for height-for-age;

*** Z BMI/A: Z-score for body mass index for age

**Table 2:** The Features of Epilepsies in Patients

**Variables**	**Weight** **(mean±SD)**	**Height** **(mean±SD)**	**BMI** **(mean±SD)**	**Zweight/A** * **(mean±SD)**	**Zheight/A** ** **(mean±SD)**	**Z BMI/A** *** **(mean±SD)**
**Predominant time of epilepsies**						
**Daytime** **Night time** **No predominance** **Total** ***P*** **-value**	26.41±11.45 32.31±9.50 30.75±10.68 29.063±10.997 *P*=0.123	125.39±15.89 134.03±12.85 129.944±15.134 128.65±15.317 *P*=0.147	16.082±2.90 17.58±2.91 17.619±2.955 16.90±2.98 *P*=0.074	0.015±0.922 -0.12±1.19 0.197±1.033 0.05±1.01 *P*=0.590	0.346±0.755 -0.39±1.300 -0.55±0.851 0.074±0.945 *P*=0.034	-0.356±1.250 0.102±1.48 0.297±1.041 -0.044±1.18 *P*=.079
**Types of epilepsy**						
**General** **Partial** **General/partial** **Total** ***P*** **-value**	30.58±11.51 27.45±10.34 22.33±5.50 29.06±10.99 *P*=0.266	130.90±15.10 125.93±15.79 122.33±10.06 128.65±15.31 *P*=0.293	17.21±3.39 16.64±2.32 14.72±1.28 16.90±2.98 *P*=0.314	0.087±1.11 0.07±0.85 -0.75±0.6 0.05±1.01 *P*=0.378	0.09±0.91 0.09±1.007 -.334±1.001 0.074±0.945 *P*=0.753	-0.014±1.273 -0.008±1.072 -0.883±0.936 -0.044±1.18 *P*=0.464
**Onset time**						
**Pearson correlation** ***P*** **-value**	0.437 0.000	0.481 0.00	0.311 0.005	0.256 0.017	0.049 0.667	0.334 0.002
**Frequency of epilepsies**						
**Pearson correlation** ***P*** **-value **	0.058 0.612	-0.058 0.607	-0.402 0.714	-0.118 0.299	-0.138 0.222	-0.011 0.926

* Z W/A: Z-score for weight-for-age;

** Z H/A: Z-score for height-for-age;

*** Z BMI/A: Z-score for body mass index for age

**Table 3:** Predicting Factors of Drug Resistant Epilepsy by Multinomial Regression Analysis

		**B**	**S.E**	**sig**	**Odds ratio**	**95% CI** **for odds ratio**	
						**Lower bound**	**Upper bound**
**Steps**	**Sex**	0.039	0.366	0.916	1.039	0.507	2.13
	**Age**	-0.003	0.008	0.742	0.997	0.981	1.014
	**Zweight/A**	-0.37	0.745	0619	0.691	0.161	2.973
	**Zheight/A**	-0.011	0.452	0.981	0.989	0.408	2.398
	**Birth weight**	-0.002	0.001	0.006	0.998	0.996	0.999
	**Birth height**	0.156	0.176	0.376	1.169	0.827	1.652
	**Gestational age**	-0.982	1.092	0.369	0.375	0.044	3.185
	**ZBMI/A**	-0.707	0.529	0.181	0.493	0.175	1.391
	**BMI**	0.23	0.173	0.183	1.259	0.897	1.767
	**Constant**	-2.85	7.724	0.712	0.058		
**Final model**	**Zweight/A**	-0.549	0.18	0.002	0.578	0.406	0.823
	**Birth weight**	-0.001	0	0.002	0.999	0.998	0.999
	**Constant**	4.484	1.386	0.001	88.617		

## Results

Thirty-two girls and 48 boys participated in each group. The majority of patients in case and control groups were term (67 (83.75%) and 72 (90%), respectively). There was no significant difference between sex groups regarding anthropometric indices Table 1) (P>0.05). (Generalized and focal types of epilepsy were noted on 57.5% and 38.75% of patients, respectively. Daytime epilepsy happened in 46.25% of patients. 33.75% of cases showed no predominant time for epilepsy. Clinicians indicated polytherapy for the majority of patients (92.5%). The most common onset times for epilepsy were 36-72 months for 32.5% of cases. Results showed lower onset time indicated lower frequency of refractory epilepsy. Furthermore, the most common frequency of epilepsies was 7-8 (86.25%). Although, there was significant difference between Zheight and predominant time of epilepsy (P<0.05), results showed no significant relation between types of epilepsy and frequency of epilepsy with anthropometric indices (Table 2). Clinicians administered sodium valproate, topiramate and priidone for most of the patients (73.75%, 35%, and 23.75%, respectively). In addition, all predicting factors of resistant epilepsy with the significant relation (P<0.10) included in multivariate regression analysis by backward LR. In final model, Zweight and birth weight were determined as the predicting factors of refractory epilepsy (Table 3).

## Discussion

In this study, we compared anthropometric indices in children with epilepsy and healthy children. Although, there was no significant difference between groups regarding height and weight but significant decreases on BMI, Z H/A, Zw/A and Z BMI/A were noted in children with epilepsy. Decreased Z-scores for H/A in this study was the same with the results mentioned by Gutheil et al. (11), that the refractory epilepsy might impair growth during childhood. However, they did not mention decreased BMI, Zw/A and ZBMI/A (11). It seems that abnormal linear growth is noted because of consuming anticonvulsant drugs and their significant effect on growth hormone concentrations but in a study, normal linear growth was noted (12). Our results showed significant relation between Zheight and the time of epilepsy. Epilepsies happened more frequently during nighttime. This might be because of growth hormone production at night, where these patients encounter with lower discharge of growth hormone. In our study, BMI in epileptic patients were less and there was significant relation between ZBMI and epilepsy. Malnutrition might decrease the threshold of epilepsy, but, more evaluation by supporting evidences from animal researches and epidemiological findings are necessary (13). Furthermore, similar to our results, Crepin et al. and Bertoli et al. observed greater risk of malnutrition in children with epilepsy than in the control group. They noted significant association between epilepsy and low BMI (3, 14). This may be because of tropical infectious diseases in which similar previous results showed that parasitic infections could invade the central nervous system and cause neurological conditions such as epilepsy (15). Therefore, it seems that further assessment regarding tropical diseases in patients with epilepsies can be recommended to enhance the patients’ treatment. Our samples had lower adipose tissues and were thinner, which might be because of hormones’ discharge from adipose cells such as leptin (16) that may decrease the threshold of epilepsies. Therefore, we recommend assessing hormones in these patients. However, leptin receptor activation, may have potential effect on anticonvulsants in emergency situations (9). Our results showed that increased birth weight reduced epilepsies threshold, which was consistent with the results mentioned previously (17, 18). However, Rocca et al. indicated no significant relation between low birth weight and the occurrence of epilepsy (19). We found no significant difference between Zweight in monotherapy and polytherapy and we could not determine the role of single treatment with topiramate in patients. However, previous investigation noted lower weight by administering topiramate (20).

**In conclusion**, deciding appropriate programs to improve the nutritional status in children with epilepsy are recommended. We recommend further investigations regarding this issue in children with epilepsy.
